# Reaching the Hispanic Community About COVID-19 Through Existing Chronic Disease Prevention Programs

**DOI:** 10.5888/pcd17.200165

**Published:** 2020-06-25

**Authors:** William A. Calo, Andrea Murray, Erica Francis, Madeline Bermudez, Jennifer Kraschnewski

**Affiliations:** 1 Department of Public Health Sciences, Penn State College of Medicine, Hershey, Pennsylvania; 2 Penn State Cancer Institute, Hershey, Pennsylvania; 3 Department of Medicine, Penn State College of Medicine, Hershey, Pennsylvania

## Abstract

Publicly available data on racial and ethnic disparities related to coronavirus disease 2019 (COVID-19) are now surfacing, and these data suggest that the novel virus has disproportionately sickened Hispanic communities in the United States. We discuss why Hispanic communities are highly vulnerable to COVID-19 and how adaptations were made to existing infrastructure for Penn State Project ECHO (Extension for Community Healthcare Outcomes) and Better Together REACH (a community–academic coalition using grant funds from Racial and Ethnic Approaches to Community Health) to address these needs. We also describe programming to support COVID-19 efforts for Hispanic communities by using chronic disease prevention programs and opportunities for replication across the country.

**Summary**

**What is already known on this topic?**

Emerging data suggest that the severe acute respiratory syndrome coronavirus 2 (SARS-CoV-2) has disproportionately affected Hispanic communities in the United States.

**What is added by this report?**

We summarize how available infrastructure from Better Together REACH, a community–academic coalition promoting chronic disease prevention, and Penn State Project ECHO, a telementoring program, was adapted to support coronavirus disease 2019 (COVID-19) pandemic efforts for the Hispanic community.

**What are the implications for public health practice?**

Leveraging resources, including community health workers, from an existing chronic disease prevention program is a promising strategy to reach Hispanic populations during these unprecedented times.

## Introduction

Pennsylvania is home to over 970,000 Hispanic people (1). Vibrant Hispanic-majority communities can be found across the state in cities such as Lebanon (total population, 25,902; 44.0% Hispanic) and Reading (total population, 88,495; 66.5% Hispanic) (1). Compared with state and national averages, incidence for Hispanic people in these 2 communities are higher for poverty, lack of health insurance, and poor health outcomes as a result of inadequate fruit and vegetable consumption, obesity, and a higher incidence of chronic diseases (2). In 2018, Better Together, a community–academic coalition led by Penn State College of Medicine, received a Racial and Ethnic Approaches to Community Health (REACH) award from the Centers for Disease Control and Prevention (CDC) to reduce the high incidence of chronic diseases among Hispanic people in both Lebanon and Reading (3). The coronavirus disease 2019 (COVID-19) pandemic has substantially affected our coalition’s ability to deliver REACH program activities because many were planned as in-person events or large community gatherings.

The pandemic has also created great fear and anxiety in Hispanic families as many face language barriers and limited access to health care and health information. The Pew Research Center recently found that about two-thirds (65%) of Hispanic adults say the novel coronavirus is a major threat to the health of the US population as a whole, compared with less than half (47%) of the general public (4). In the same national survey, more Hispanic adults than American adults overall also said that COVID-19 is a major threat to their personal health (39% vs 27%, respectively) (4). Recognizing these challenges, our REACH coalition has strategically shifted resources to actively support the demands of local and state COVID-19 response efforts while still attending to our main goal to reduce disparities related to chronic disease prevention. The objective of this commentary is to discuss why Hispanic communities seem to be highly vulnerable to COVID-19, summarize the Better Together REACH initiatives, discuss how Better Together REACH has adapted program offerings to support COVID-19 pandemic efforts for the Hispanic community, and consider steps that might be taken to replicate these efforts across the country.

## Hispanic Communities Are Especially Vulnerable to COVID-19

Publicly available data on racial and ethnic disparities related to COVID-19 (ie, people who have been tested for, who were infected by, or who have died from the virus) are now surfacing, and these data suggest that the novel virus has disproportionately sickened Hispanic communities (5–7). For example, in Pennsylvania’s neighboring state New Jersey, 19% of the total population is Hispanic but Hispanic people make up 30% of COVID-19 cases (6). Similar COVID-19 case rate disparities for Hispanic people are reported in many states across the United States such as Utah (14% of total population vs 38% of COVID-19 cases) and Washington (13% of total population vs 34% of COVID-19 cases) (6). Partial COVID-19 death data disaggregated by Hispanic ethnicity also show that Hispanic people are dying at a rate above what population data would suggest (7). For example, CDC’s weighted population data show that over 26% of US COVID-19 deaths were among Hispanic people, who represent only 18% of the total US population (7). In Pennsylvania, where Hispanic people are 7.6% of total state population, 11% of COVID-19 deaths were among Hispanic people, when applying weighted population distributions (7).

The vulnerability of Hispanic communities to COVID-19 can arise from many factors, including differential exposure, susceptibility, and access to health care (8). First, many Hispanic people work in frontline jobs in grocery stores, waste management, cleaning and sanitation services, and food delivery (9), putting them at constant exposure to people or materials that may be infected with COVID-19 (10). In addition to work circumstances, living conditions may also increase exposure to COVID-19 among Hispanic families (11). Twenty-five percent of Hispanic people live in multigenerational households (compared with only 15% of non-Hispanic white people) (12), which may make it challenging to take precautions to protect older family members or to isolate those who are sick if space in the household is limited (11). Although having a chronic disease does not increase the risk of contracting the new coronavirus, the presence of chronic disease can worsen the outcome of COVID-19 (13). Emerging data from the state of New York show that among those who died of COVID-19 (23,083 people as of May 20, 2020), the leading underlying illnesses were hypertension (54% of deaths) and diabetes (36% of deaths) (5). This is alarming for Hispanic people because they have higher rates of both hypertension and diabetes as compared with non-Hispanic white people (14). Also, the lack of reliable information in Spanish has impeded efforts to combat the spread of the virus in Hispanic communities (15). This is especially true among those with language barriers, making them more likely to be unaware of best practices. Lastly, Hispanic people are the largest population segment without health insurance coverage in the United States (14), leaving those with presumptive symptoms or with a positive COVID-19 test with limited access to needed health care.

## Better Together REACH Initiatives

Established in 1999, REACH is CDC’s cornerstone program aimed at reducing racial and ethnic health disparities. In 2018, CDC funded a new 5-year cycle of 31 grant recipients to reduce health disparities among racial and ethnic populations (ie, Hispanics, African Americans, American Indians, Asian Americans, Alaska Natives, and Pacific Islanders) with the highest level of chronic disease such as hypertension, heart disease, type 2 diabetes, and obesity (3). Through REACH, recipients plan and carry out local, culturally appropriate programs to address preventable risk behaviors leading to chronic diseases, such as poor nutrition and physical inactivity. Given the overwhelming socioeconomic and health disparities that Hispanic people face in both Lebanon and Reading, our coalition focused on improving chronic disease prevention outcomes in these 2 communities. Since 2018, Better Together REACH has leveraged strong community collaborations to implement locally tailored practice-based and evidence-based strategies aimed at increasing healthy nutrition programming, physical activity opportunities, and diabetes prevention programs. This initiative brings together over 60 local organizations that are now working together to break down silos, to share a common agenda to address health disparities, and to improve community wellness and the quality of life for all their residents (16).

Two of our signature initiatives related to healthy nutrition include expanding access to affordable and nutritious food (eg, Farmers Market Nutrition Program; Veggie Rx, a fruit and vegetable prescription program to alleviate food insecurity among patients with diabetes) and creating bilingual hospital-based breastfeeding programming and support with local Special Supplemental Nutrition Program for Women, Infants, and Children (WIC) offices. To improve physical activity opportunities, we are actively promoting use of existing walking and bike routes that connect everyday destinations (eg, parks, schools, businesses, community facilities) and supporting the planning and designation of new routes (eg, Walk Works). To address critical community–clinical linkages, we are expanding access to diabetes prevention program offerings by training local, bilingual community health workers (CHWs) to connect at-risk people with existing programs and supporting the CHWs to become certified lifestyle coaches. Our initiatives are promoted throughout our community networks with culturally relevant marketing campaigns. Many of these initiatives have been paused following CDC’s social distancing recommendations and Pennsylvania’s stay-at-home orders. What has not paused in the face of the pandemic is the commitment of our coalition to serve the Hispanic communities in Lebanon and Reading in these uncertain times. Our local and state partners are now facing an increased demand for health and social services, without receipt of additional resources and while simultaneously experiencing a loss of revenues and staff. The Better Together REACH team has been quick to recognize these challenges and the changing needs of the Hispanic communities over the past few months.

## Local Response to Help Hispanic Communities

Since Pennsylvania’s Department of Health confirmed the first cases of COVID-19 in early March, the Better Together REACH team has been working to assist the Lebanon and Reading communities in their fight against this novel disease. As the rapidly evolving pandemic unfolds across our communities, families are faced with unprecedented challenges including loss of income, which has a trickledown effect in their ability to support basic needs. National survey data show that Hispanic adults (44%) were more likely than non-Hispanic white adults (26%) to report that they “cannot pay some bills or can only make partial payments on some of them” as a result of the economic challenges caused by the pandemic (17). Sixty-one percent of Hispanic adults also reported that they or someone in their household had lost a job or wages because of the coronavirus pandemic, compared with 38% of non-Hispanic white adults (17). Many members of our community are unpaid if their employers cannot open for business, and those who are immigrants are less likely to qualify for most government-sponsored assistance programs. Acknowledging these major issues, our team developed and disseminated a 1-page resource in Spanish to address questions about emergency lodging, food access, unemployment benefits, utility payments, and other nonmedical basic needs in Lebanon and Reading during local COVID-19 response events. This resource has been distributed to families picking up meals from local school district distribution sites.

Our team also identified, and has helped to address, the need for Hispanic families to stay informed about best practices to avoid the spread of COVID-19 as well as how and where to seek testing and health care if they develop symptoms. To better understand this need, our bilingual CHW convened virtual meetings with Hispanic community leaders and organizations serving Hispanic people (Figure). Through these conversations, we learned that Hispanic people were struggling to access reliable information in Spanish. We also learned that many had access to smartphones and internet (major carriers are now providing free internet access during the pandemic), and they were willing to join remote learning activities if offered in Spanish. With this information in hand, we reached out to Penn State Project ECHO (Extension for Community Healthcare Outcomes) to facilitate a series of community-facing webinars in Spanish to disseminate information about COVID-19.

**Figure:**
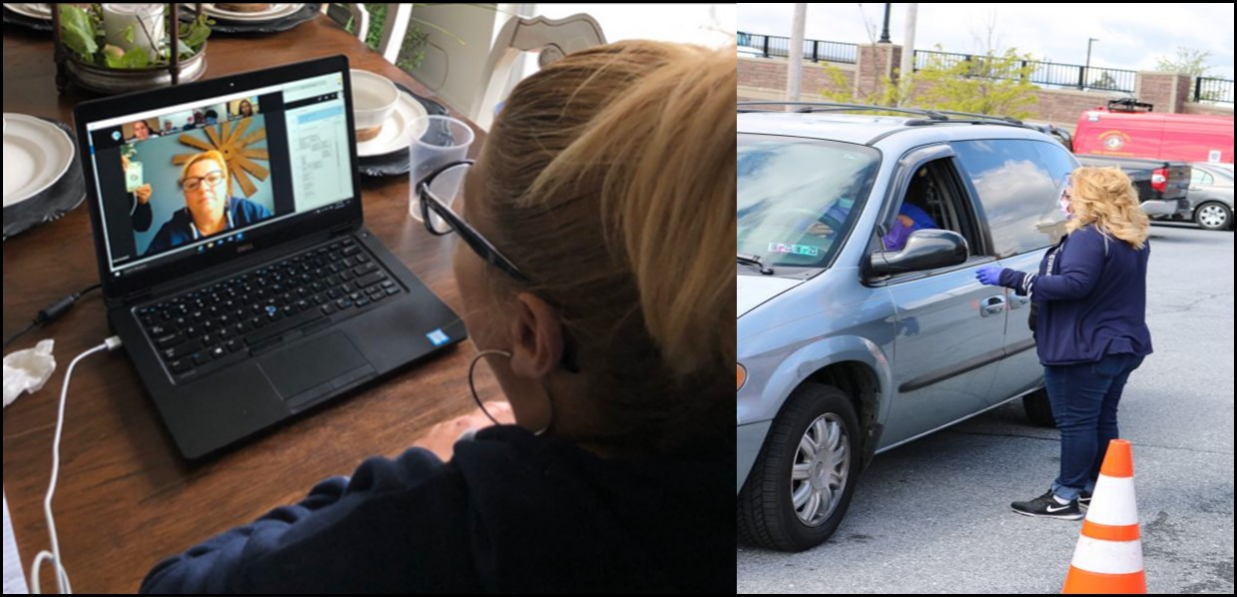
Community health worker leading a video conference call with community leaders in Lebanon County, Pennsylvania, to discuss the needs of Hispanic residents regarding coronavirus disease 2019 (COVID-19) (left) and distributing masks, bottles of hand sanitizer, and Spanish-language public service announcements at a local drive-through COVID-19 response site (right).

We partnered with Penn State Project ECHO at the right time, as they had launched a COVID-19 ECHO series on March 20 to inform health care providers and administrators of the latest best practices in emergency preparedness and patient treatment of COVID-19. Through this series of 1-hour webinars, participants presented patient and clinic or hospital system cases to academic expert teams who mentor them on patient care and systems quality improvement. These case-based discussions were supplemented with brief didactic presentations to improve content knowledge and share evidence-based best practices for dealing with COVID-19. Project ECHO is not telemedicine where expert specialists assume the care of the patient, but instead is “telementoring.” Registered participants received the sessions via real-time, interactive videoconferencing by using Zoom (https://zoom.us; Zoom Video Communications, Inc), a user-friendly, Health Insurance Portability and Accountability Act (HIPAA)–compliant, cloud-based software application offered at no cost to them. Zoom has numerous benefits, including the ability to run on lower-speed internet connections. Participants easily connected to sessions by using a PC or Mac computer, laptop computer, tablet, or smartphone with or without a camera.

By using this existing infrastructure, we conducted the first Spanish-language community-facing COVID-19 ECHO series for the Hispanic community on April 2. This first session was “Preparing your household for COVID-19” and it was well attended through Zoom with concurrent transmission via Facebook Live (Table). While our bilingual CHW continued communicating with Hispanic community leaders daily, she assessed the evolving needs of Hispanic people in Lebanon and Reading with regard to COVID-19. Knowing our community needs, we planned and delivered Spanish-language sessions on diabetes management, mental health resources for families, and how to keep children physically active and eating healthily during COVID-19 times. We partnered with bilingual health care providers and public health scientists with expertise on these topics to deliver the sessions in Spanish. Additional sessions are being scheduled for upcoming weeks (eg, the role of CHWs in COVID-19 responses).

**Table:** Participation Metrics of the Spanish-Language Community-Facing COVID-19 Project ECHO Series, Lebanon and Reading, Pennsylvania, 2020

Session Topic	Zoom^a^	YouTube^b^	Facebook		
			Reach^c^	Engagement^d^	Views^e^
Preparing your household for COVID-19^f^	62	250	746	15	122
Diabetes management^g^	36	122	1,595	209	509
Mental health resources for families^h^	31	90	415	92	165
Healthy eating and physical activity^i^	24	54	258	30	140

Abbreviation: COVID-19 Project ECHO, Coronavirus disease 2019 Project ECHO (Extension for Community Healthcare Outcomes).

a Number of unique people who joined the session via Zoom (https://zoom.us; Zoom Video Communications, Inc).

b Number of recording views in YouTube as of May 27, 2020 (https://www.youtube.com; YouTube, LLC).

c Number of unique people (estimated metric) who saw any session content in Facebook (https://www.facebook.com; Facebook, Inc).

d Total number of actions (eg, likes, comments, shares) that people took involving the session.

e Number of times the session content was viewed by people.

f Spanish title shown in YouTube “COVID-19: Estrategias para preparar su hogar y cuidar a su familia” (April 2, 2020).

g Spanish title shown in Youtube “COVID-19: Manejo de la Diabetes” (April 14, 2020).

h Spanish title shown in YouTube “COVID-19: Recursos de salud mental para familias durante la pandemia” (April 22, 2020).

i Spanish title shown in YouTube “COVID-19: Como Mantener Niños Activos y con Habitos Alimentarios Saludables en tiempos de COVID-19” (May 6, 2020).

A key feature of our community-facing COVID-19 ECHO series was the opportunity for community members to actively participate in discussions about situations or challenges they have faced. Before each session, our CHW assessed questions or concerns from the community, so speakers used that information to craft their presentations and discuss those real-world scenarios as de-identified cases. These local cases served to reinforce the didactic portion of the webinar. Because we used an “all teach, all learn” approach, community members were free to ask questions and participate in discussions at any time during the session. Participants had the option to write questions in the chat box or use the raise hand feature to indicate that they had a question or comment to share with all participants. We also instructed the presenters to set aside the last 10 to15 minutes of the session to allow questions from the public. Because these community-facing sessions were delivered in Spanish, all questions were raised and responded to in the same language. Most of the participants’ comments were requests for educational materials in Spanish to be distributed in their communities.

After each session, we made available to the general public the video recordings through the Penn State Project ECHO’s YouTube channel (https://bit.ly/COVID_Spanish; YouTube, LLC). Presentation slides and other resources (eg, information sheets from CDC, public service announcements [PSAs] developed by Better Together REACH [18]) discussed in the sessions were also sent to participants via email or shared through access to a dedicated online shared folder. The success of our community-facing COVID-19 ECHO series motivated other collaborators to launch a Nepali-language series to reach the growing Nepali Bhutanese community that has found refuge in Pennsylvania. Our Better Together REACH team also supported the COVID-19 ECHO series for health care providers and administrators by organizing and presenting sessions about maternity health and breastfeeding and how to reach minority populations during the pandemic.

We have also been very active supporting local organizations and state agencies in their communication efforts. The Pennsylvania Commission on Latino Affairs and the state’s Office of Health Equity have noted a lack of reliable messaging in Spanish about COVID-19 as a barrier for information dissemination in the state. To address this issue, we have translated health communication materials for local nonprofit organizations needing assistance in serving Spanish-speaking Hispanic people, and we have created educational resources in Spanish to help families stay informed during the pandemic. For example, we developed a collection of Spanish and English PSAs, which have been published through regional media outlets and distributed at local events to reinforce the importance of following CDC guidelines for preventing the spread of COVID-19. We developed these PSAs with an understanding that not all community members have access to a computer or internet in their homes. These PSAs are available for any community organization to use and can be freely accessed online (18), already having been shared with the National REACH Coalition.

## Opportunities for Next Steps

As we did with the existing infrastructure of Better Together REACH, other chronic disease prevention programs can employ similar promising strategies to reach vulnerable populations across the country during these unprecedented times. Using the infrastructure of Penn State Project ECHO to deliver Spanish language, community-facing webinars was an invaluable asset to connect hard-to-reach populations with best-practice communication about COVID-19. Equally important, supporting our COVID-19 responses with CHWs was effective for both public health and community well-being.

We need to continue leveraging available infrastructure and technology to amplify the unique community connections CHWs have. On the basis of our own experience in Pennsylvania, we can offer several suggestions, although we acknowledge that every community faces unique challenges and every organization has unique strengths and limitations. We found that CHWs can easily use low-key and freely available technology like Zoom or social media to get real-time data from local leaders and organizations and share it with decision makers so that they can disseminate health and social service resources to vulnerable populations. CHWs can likewise deliver evidence-based information about COVID-19 prevention, testing, and health services to community members. At a time when misinformation is widespread and culturally appropriate information is limited, CHWs’ communication skills are more important than ever. Also, as many health care organizations and government health agencies are turning to CHWs to fill gaps in community-based pandemic response efforts, including contact tracing, we have to protect their well-being (19).

## Implications for Public Health

Despite the observed health disparities, the pandemic presents a window of opportunity for achieving greater equity in preventing disease and providing health care for vulnerable populations (20). To achieve this goal, we require improved data collection to monitor and track disparities among racial and ethnic groups in the number of COVID-19 cases, complications, and deaths (20). These data would serve to quickly inform decisions on how to effectively address disparities and allocate resources at different levels of action. We also need consistent and credible culturally appropriate information to share with the general public (11,15). CHWs are proven to be effective messengers (19). Increasing the CHW workforce, especially in underserved communities, can meet the urgent demand to educate and connect people to health care services (19). Efforts should continue working across sectors beyond health to identify critical resources, such as temporary housing, because many families are now facing serious financial struggles (11). Our experience suggests that by working together, we all help to make our communities stronger, more stable, and healthier.
